# Telomerase Reverse Transcriptase (TERT) Regulation in Thyroid Cancer: A Review

**DOI:** 10.3389/fendo.2020.00485

**Published:** 2020-07-31

**Authors:** Brittany A. McKelvey, Christopher B. Umbricht, Martha A. Zeiger

**Affiliations:** ^1^Department of Surgery, Johns Hopkins University School of Medicine, Baltimore, MD, United States; ^2^Department of Molecular Biology and Genetics, Johns Hopkins University School of Medicine, Baltimore, MD, United States; ^3^Department of Oncology, Johns Hopkins University School of Medicine, Baltimore, MD, United States; ^4^Department of Pathology, Johns Hopkins University School of Medicine, Baltimore, MD, United States; ^5^Surgical Oncology Program, National Cancer Institute, National Institutes of Health, Bethesda, MD, United States

**Keywords:** *TERT*, telomerase, thyroid cancer, transcription, epigenetics, copy number variation, alternative splice variants

## Abstract

*Telomerase reverse transcriptase (TERT)* is the catalytic subunit of the enzyme telomerase and is essential for telomerase activity. Upregulation of *TERT* expression and resulting telomerase activity occurs in the large majority of malignancies, including thyroid cancer. This upregulation results in continued cellular proliferation and avoidance of cellular senescence and cell death. In this review we will briefly introduce *TERT* and telomerase activity as it pertains to thyroid cancer and, highlight the effects of *TERT* on cancer cells. We will also explore in detail the different *TERT* regulatory strategies and how *TERT* is reactivated in thyroid cancer cells, specifically. These regulatory mechanisms include both activating single base pair *TERT* promoter mutations and epigenetic changes at the promoter, including changes in CpG methylation and histone modifications that affect chromatin structure. Further, regulation includes the allele-specific regulation of the *TERT* promoter in thyroid cancer cells harboring the *TERT* promoter mutation. These entail allele-specific transcriptional activator binding, DNA methylation, histone modifications, and mono-allelic expression of *TERT*. Lastly, *TERT* copy number alterations and alternative splicing are also implicated. Both amplifications of the *TERT* locus and increased full-length transcripts and decreased inactive and dominant negative isoforms result in active telomerase. Finally, the clinical significance of *TERT* in thyroid cancer is also reviewed.

## Introduction

In contrast to stem cells, non-transformed somatic cells have a limited capacity to divide in tissue culture before cell division ceases. This is known as cellular senescence and the number of times a normal human somatic cell population will divide before cell division stops is referred to as the Hayflick limit (1). Cellular senescence results from the progressive shortening of chromosomal ends or telomeres, consisting of identical hexamer repeats, with each cell division. This phenomenon is due to the end replication problem, a shortcoming of semiconservative DNA replication, which cannot complete the synthesis of chromosomal ends (2). This critical shortening of telomeres continues until reactivation of the enzyme, telomerase, facilitates the resynthesizing of telomeres of sufficient length to allow continued cellular replication. Telomere lengthening can also occasionally occur through the alternative lengthening of telomeres (ALT) pathway, which extends telomeres without telomerase activity through recombination-dependent pathways. The ALT pathway remains poorly understood and will not be covered in this review of *TERT* regulation (3).

Telomerase is composed of two subunits: an RNA component, telomerase RNA (TR), serving as a template for telomere hexamer repeat addition onto DNA and, the catalytic component telomerase reverse transcriptase (TERT), responsible for reverse transcribing the hexamer repeats onto chromosomal ends (4). Not only is telomerase active in embryonic and stem cells, it is also upregulated in over 90% of malignancies, including thyroid cancer, enabling the unlimited replication of cancer cells (5–8).

*TERT* activation in cancer occurs through a variety of mechanisms. These include activating promoter mutations, alterations in promoter DNA methylation, chromatin remodeling, copy number alterations, and alternative splicing of *TERT* (9–13). Because telomerase plays a key role in carcinogenesis, understanding *TERT* regulation and telomerase activation in thyroid cancer is critical to understanding its pathogenesis. Indeed, two *TERT* regulatory mechanisms, *TERT* promoter mutations and *TERT* promoter methylation status, are implicated in the stratification of thyroid cancer patient prognosis (14–16).

In this review, we summarize our current understanding of the multiple mechanisms of *TERT* regulation in thyroid cancer. Activating point mutations in the *TERT* promoter markedly activate *TERT* transcription and are associated with epigenetic alterations observed in thyroid cancer cell lines and patient tumors. These epigenetic alterations include promoter methylation patterns and histone tail methylation modifications. The modifications have also been shown to affect the *TERT* promoter in an allele-specific manner, the details of which we will elucidate. Further, the *TERT* locus has also been found to have multiple chromosomal copies or, be amplified in cancer with associated increased *TERT* transcription. Lastly, post-transcription, the *TERT* transcript undergoes alternative splicing to form either the full-length transcript, which is the active isoform, or other inactive isoforms, which may also have regulatory functions. Finally, we highlight the clinical implications of *TERT* activation and, describe future potential avenues to explore in order to better understand regulation of this key enzyme as well as its role in carcinogenesis and cancer prognosis.

## *TERT* Promoter Mutations

**Figure 1:**
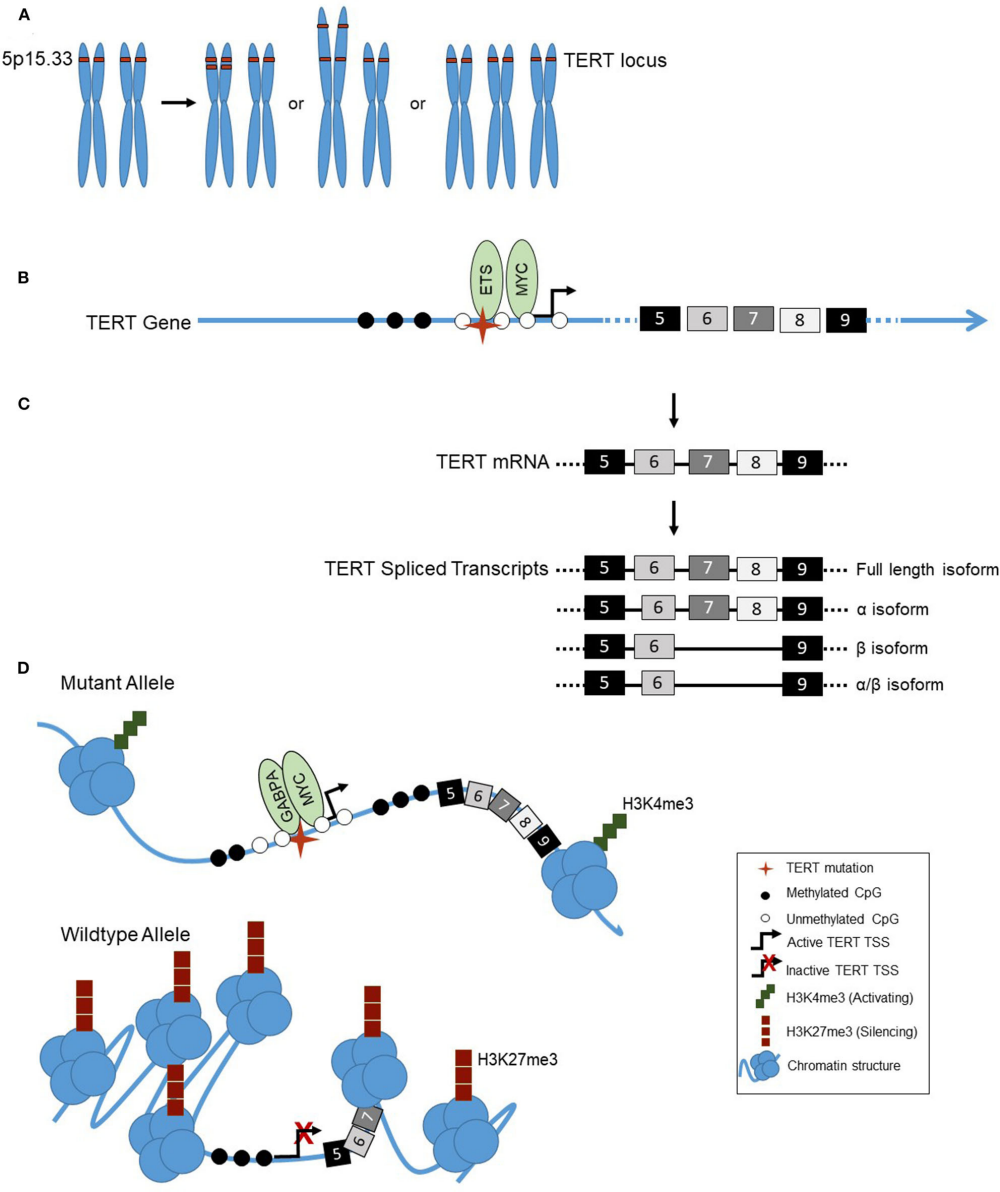
**(A)** Examples of TERT copy number amplifications (chromosomal location 5p15.33): Focal copy number alterations, arm-level alterations, or chromosomal duplications (trisomy 5). **(B)** The landscape of the TERT promoter in thyroid cancer shows a lack of methylation (◯) surrounding the transcription start site, TSS, (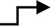) and TERT promoter mutation (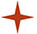), with hypermethylation further upstream of the TSS (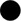). Transcriptional activators MYC and ETS family factors bind at the TERT promoter, the latter binding in the presence of the TERT mutation only. **(C)** Alternative splicing of TERT in thyroid cancer. TERT is transcribed into mRNA, containing 16 exons (#5–9 shown). TERT can be alternatively spliced, into either the full length, α, β, or α/β isoforms. **(D)** Allele-specific regulation of the TERT promoter in thyroid cancer. The TERT mutant allele, with mutation (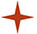) is in an open chromatin conformation, with histones spread across the DNA, depicted by the cluster of blue circles. The wildtype allele is in the closed chromatin conformation, with compacted histones on the DNA. The mutant TERT allele is associated with activating H3K4me3 histone marks, represented by the green squares, a CpG unmethylated (◯) promoter and methylated (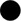) gene body. The transcription factors GABPA and MYC bind at the promoter, and TERT is actively transcribed. The wildtype allele is associated with silencing H3K27me3 histone marks, represented by red squares, and a CpG methylated promoter.

Activation of *TERT* transcription can be achieved by heterozygous point mutations at the *TERT* promoter. The two most common activating mutations are upstream of the *TERT* translation start site at −124 and −146, respectively, and both are cytosine to thymine mutations, −124C>T and −146 C>T (Figure 1B) (17–19). These *TERT* promoter mutations are not present in benign thyroid tumors or normal thyroid cells. Their prevalence, however, increases with more aggressive thyroid cancer subtypes and advanced stages of disease. Indeed, *TERT* mutations are present in only 11.3% of well-differentiated papillary thyroid cancer (PTC) and 17.1% of follicular thyroid cancer (FTC), but present in 32% of widely invasive Hürthle cell carcinoma (HCC), compared to 5% of minimally invasive HCC, and in 43.2% of poorly differentiated thyroid cancer (PDTC) and 40.1% of anaplastic thyroid cancer (ATC) (20, 21). Furthermore, the *TERT* promoter mutations are found in almost all thyroid cancer cell lines, even those derived from well-differentiated thyroid cancer (DTC) subtypes, in either the heterozygous or homozygous mutant state, indicative of their important role in cellular proliferation (11).

As seen in the TCGA data, the presence a *TERT* mutation is also strongly associated with high risk of tumor recurrence, older age, higher MACIS (Metastasis, patient Age, Completeness of resection, local Invasion, and tumor Size) scores, and less-differentiated PTC (22). In support of the TCGA data, in a large study of thyroid cancers in which anaplastic carcinoma coexisted with papillary carcinoma, a multivariate comparison between the antecedent papillary carcinoma components and control papillary carcinomas without anaplastic transformation showed that *TERT* mutations were independently associated with anaplastic transformation (23). Further studies have also highlighted the association of the *TERT* promoter mutation and non-radioiodine avidity of metastatic disease (24, 25).

### Mechanism of *TERT* Mutation Activation

Likely key to their effect, both *TERT* promoter mutations result in a novel 11 base pair binding site for the E-twenty-six (ETS) transcription factor family. The −124C>T mutation, however, elicits a higher activation of *TERT* compared to the −146C>T mutation and, is more common (20, 26). The activating GA Binding Protein Transcription Factor Subunit Alpha (GABPA), a member of the ETS family, binds at the *TERT* promoter mutation site as a heterotetramer with its counterpart, GABPB, in multiple cancer types, including thyroid cancer (26–29). In PTC cell lines, *GABPA* knockdown significantly lowered *TERT* expression in both wildtype and *TERT* promoter mutant cells, and in a luciferase activity assay, *GABPA* knockdown led to significant down regulation of the mutant *TERT* promoter. However, in an analysis of papillary thyroid cancers and TCGA data, an inverse relationship was found between *GABPA* expression and *TERT* expression in *TERT* promoter mutant tumors, highlighting the need for further study of *TERT* mutation activation (30).

Recently, another ETS factor, ETS Variant 5 (ETV5), was also found to bind the mutant *TERT* promoter and, bound at higher levels than GABPA in ATC cell lines. Conversely, GABPA bound at higher levels than ETV5 in well-differentiated thyroid cancer-derived cell lines (31), implicating potentially different roles of these specific ETS factors in well-differentiated and poorly differentiated cancers.

### Synergistic Effects of *TERT* and *BRAF*V600E Mutation

The *BRAF*V600E mutation is also common in thyroid cancer. The *BRAF*V600E mutation is a single nucleotide mutation at codon 600, resulting in a substitution of glutamic acid for valine in the BRAF protein, a serine/threonine protein kinase that plays a role in the mitogen-activated protein kinase (MAPK) pathway/ERK signaling pathway. The *BRAF*V600E mutation is more abundant in classic PTC (51%) than in the follicular variant of PTC (FVPTC, 24%) or FTC (1.4%) (32). The *BRAF*V600E mutation acts synergistically with *TERT* promoter mutations and the presence of both mutations is associated with greater cancer aggressiveness, lymph node and distant metastasis, advanced tumor stage, recurrence, and increased mortality in patients with PTC (33–35). The mutation may also be responsible for increased activation of the ETS factor family through activation of the MAPK pathway. There are two prevailing proposed mechanisms for the activation of *TERT* by the *BRAF*V600E mutation and, the MAPK pathway. In one study of PTC cell lines, the MAPK pathway activates the transcription factor c-FOS, which can in turn bind to the *GABPB* promoter to increase its expression. This then leads to increase in GABPA-GABPB complex formation, which binds to the mutant *TERT* promoter to activate *TERT* (27). Alternatively, another study in PTCs from the TCGA study cohort shows upregulation of ETS factors ETV1, ETV4, and ETV5 in tumors that concomitantly harbored both *BRAF*V600E and *TERT* mutations and, demonstrated MAPK pathway activation. In cell lines, these ETS factors were found to bind to the mutant *TERT* promoter and upregulate transcription (36).

## *TERT* Epigenetic Alterations

Epigenetic changes are stably inherited during cell division and occur at DNA or chromatin levels, but do not alter the primary base sequence (37). At the DNA level, epigenetic alterations occur by methylation of the cytosine nucleotide in cytosine-guanine dinucleotides (CpGs). CpG methylation surrounding the transcriptional start site (TSS) at gene promoters is usually associated with suppression of gene expression. Conversely, the absence of DNA methylation surrounding the TSS of genes is associated with active transcription. Transcription factors that bind and regulate gene promoters can also be methylation sensitive, where methylation impedes binding (38). The *TERT* promoter is within a CpG island and has a rich GC content of ~70% (39). Previous studies in a variety of cancer cell lines as well as solid tumors and hematological malignancies show a unique methylation pattern of *TERT*. Its promoter region surrounding the TSS [−200 to +100 relative to TSS] is unmethylated, while the upstream promoter region [−650 to −200] is hypermethylated (Figure 1B) (13, 40). The unmethylated TSS allows for transcription to occur by availing its binding sites for activators, such as MYC, and when the *TERT* promoter mutation is present, ETS family factors (41). The role of the hypermethylated upstream promoter, and its association with *TERT* expression, however, is unknown.

### *TERT* Methylation in DTC

Our recent study in DTC cell lines and normal thyroid tissue confirmed that this dual methylation pattern observed in other cancer types is also seen in thyroid cancer (28). Five DTC cell lines exhibited low levels of methylation surrounding the *TERT* TSS while the upstream region was largely methylated. Comparatively, normal thyroid tissue showed overall low levels of methylation throughout the *TERT* promoter compared to the thyroid cancer cell lines (28, 29). Ours is the only study to date that comprehensively characterized *TERT* promoter methylation, as previous studies have focused on the hypermethylated upstream region only. A 2018 study of follicular neoplasms found the upstream *TERT* promoter was methylated significantly higher in FTC (13%) than follicular adenoma (8%) (42). Lastly, a study of upstream *TERT* promoter methylation patterns in 312 PTC, FTC, MTC, and ATC patients found significantly higher upstream DNA methylation in patients with cancer recurrence than in patients whose tumors did not recur. In patients with a recurrence, upstream *TERT* promoter methylation was also associated with higher tumor stage and the presence of lymph node metastasis (15).

### *TERT* Upstream Methylation in MTC

A 2016 study of medullary thyroid cancer (MTC) and normal thyroid tissue samples quantified the *TERT* upstream promoter methylation at eight CpG sites. Sporadic MTCs had a significantly higher methylation level (12–90.3%) compared to normal thyroid tissues (~10%). And, this methylation pattern positively correlated with expression of *TERT* and telomerase activation. Further, high methylation of the upstream *TERT* promoter correlated with shortened survival in patients with MTC (16).

### *TERT* Histone Mark Modifications

In addition to methylation aberrations, epigenetic changes in the histone code can also modify gene expression by altering the chromatin state from a closed, inactive state to an open, actively transcribed state. The histone mark H3K27me3 maintains heterochromatin and recruitment of the polycomb repressor complex (PRC) for silenced genes (43). Alternatively, the H3K4me3 mark maintains actively transcribed genes in the open confirmation to enable access for transcriptional activators to bind (44). Telomerase negative primary cells exhibit high levels of the silencing H3K27me3 mark at the *TERT* promoter, compared to telomerase positive cancer cell lines (45), which exhibit the activating H3K4me3 mark (46).

This histone mark pattern was also seen in our recent study. The activating mark H3K4me3 was least abundant in the benign thyroid-derived cell line N-thy-ori-3, as well as a cell line wild type for the *TERT* mutation. There were intermediate levels of the mark in the cell lines heterozygous for the *TERT* promoter mutation, and most abundant in the homozygous *TERT* mutant cell line. The silent mark H3K27me3 displayed the opposite pattern, showing the highest level in the normal and wildtype thyroid cell lines, intermediate in the heterozygous mutant cell lines, and least abundant in the homozygous mutant cell line. The latter phenomenon depicts a pattern of activation at the *TERT* promoter in cancer cells that is dependent on the *TERT* promoter mutation status, where activation by chromatin marks also correlated with *TERT* expression (29).

## Allele-Specific Regulation

Recently, in order to further delineate *TERT* transcriptional regulation, studies have been conducted in many cancer subtypes to examine allele-specific regulation and the *TERT* mutation (Figure 1D). Allele-specific regulation, resulting in expression of only one allele, is classically described by the well-known phenomenon, inactivation of one of the X chromosomes in all females. This is due to epigenetic effects, in which one of the two X chromosomes is stably transcriptionally silenced by methylation early in development (47).

A study in 2015 of multiple cancer cell lines showed monoallelic expression of *TERT* in cell lines heterozygous for the *TERT* promoter mutation, although the study did not elucidate which allele was transcribed (48). Further studies expanded upon this by showing allele-specific regulation of the alleles, in which the *TERT* promoter mutant allele exhibits the H3K4me2/3 activating histone modification as well as binding of RNA polymerase II, the polymerase responsible for actively transcribing genes. The activating ETS factor GABPA has also been shown in multiple cell lines to bind in an allele-specific manner to the mutant *TERT* promoter only. This would be expected since the mutation specifically creates the ETS factor binding site. The wild type *TERT* allele, however, displays the silencing histone mark H3K27me3 and, is associated with the PRC (45, 49).

### *TERT* Allele-Specific Regulation in Thyroid Cancer

Allele-specific regulation in thyroid cancer cell lines has been explored by our group and others. In our recent study of five DTC cell lines we found the mutant allele at the *TERT* promoter was significantly less methylated than the wildtype allele, while the corresponding gene body associated with the mutant allele was more methylated. Further, a significant drop in methylation at the TSS of the mutant allele, but not the wildtype allele, was observed in the three heterozygous mutant cell lines. The methylation pattern observed on the mutant allele, of decreased methylation at the TSS and increased gene body methylation, is seen in actively transcribed genes. Additionally, we found that the activating transcription factors MYC and GABPA, bound to the mutant *TERT* allele exclusively. As expected, the activating histone mark H3K4me3 was associated with the mutant allele while the silenced mark H3K27me3 was associated with the wildtype allele. Bullock et al.'s recent study of two heterozygous mutant thyroid cancer cell lines suggested allele-specific regulation of *TERT*, showing monoallelic expression of *TERT* in the ATC and PTC cell lines. In the cell lines H3K4me3 was associated with the mutant allele only, while a majority of H3K27me3 was associated with the wildtype allele. In one ATC line, the ETS factor ETV5 bound in an allele-specific manner (31). Furthermore, by exploiting the presence of heterozygous SNPs in the transcripts, our group has definitively demonstrated that this allele-specific regulation results in selective transcription from only the mutant *TERT* promoter (29).

## *TERT* Alternative Splicing

Previous studies have shown *TERT* expression is undetectable in normal thyroid tissue, low to absent in benign tumors, and elevated in thyroid cancers (20). *TERT* promoter mutations also are significantly associated with increased levels of *TERT* expression (20, 50). In addition, gene expression regulation can occur through alternative splicing (51). Indeed, *TERT* is regulated by alternative splicing, and thought to be crucial because even small amounts of *TERT* may have significant cellular consequences (Figure 1C) (9). This is exemplified by the fact that telomerase-positive malignant cells are estimated to contain only an average of 20 *TERT* transcripts per cell (52). The most common *TERT* isoforms include the following: (1) full-length transcript, the only isoform resulting in active telomerase (53, 54); (2) the α deletion isoform, an in-frame deletion of 36 base pairs in exon six resulting in a dominant negative inhibitor of telomerase (55); (3) the β deletion isoform, a 182 base pair deletion of exons seven and eight resulting in a reading frame shift forming a truncated *TERT* transcript that is sent for nonsense mediated decay (56); and (4) the α/β dual deletion isoform. Studies of *TERT* alternative splicing in multiple cancer types have shown the highest expressed isoform in cancer is the full-length isoform, which correlates with higher telomerase activity (57–59).

### *TERT* Splicing in MTC

A study of 42 MTC tumors found *TERT* expression and telomerase activity in 50% (21/42) of the tumors. And, over two-thirds of the telomerase positive cancers (15/21) showed expression of the full-length *TERT* transcript, higher telomerase activity and were associated with a significantly shorter patient survival time (60).

### *TERT* Splicing in DTC

Our group showed, in a study of 60 malignant tumors (PTC, FVPTC, FTC, and HCC) and 73 benign lesions, that malignant tumors showed a higher amount of the full-length isoform than the inactive *TERT* isoforms, while conversely, the benign tumors showed higher levels of the deletion isoforms (61). Further, only the full-length transcript correlated with telomerase activity (61, 62). By computing the proportion of full-length isoform to the deletion isoforms, we found the percentage score sorted thyroid tumors into subtypes; with the high full-length fractions of *TERT* found in PTC, HCC, and FTC, while intermediate fractions were found in FVPTC, Hürthle cell adenomas, and follicular adenomas and finally, low fractions in adenomatoid nodules (62).

## *TERT* Copy Number Amplifications

DNA copy number variations, the gain or loss of chromosomal regions, including whole chromosomal arms (Figure 1A) within the genome, are quite common in cancer. They can provide a tumor cell proliferative advantages by eliminating tumor suppressors, in the case of deletions, or increasing expression of oncogenes, in the case of amplifications. *TERT* is located on the short arm of chromosome five (5p). 5p is one of the most frequent arm level regions to exhibit amplification with the phenomenon found in 13.2% of solid tumors (63). Further, a variety of high resolution techniques used to examine the *TERT* locus specifically, including fluorescence *in situ* hybridization (FISH), Southern blot, and quantitative PCR, have shown copy number amplifications of *TERT* in both cancer cell lines and tumors (63). Studies have also documented a correlation in cell lines of increased *TERT* copy number with both increased *TERT* gene expression and telomerase activity (64).

A recent study by Panebianco et al., of 184 tumors of various thyroid cancer subtypes- PTC, FTC, HCC, MTC, and PDTC/ATC, identified an increased *TERT* copy number in 4.9% of tumors (65). Specifically, 1/107 (0.9%) PTCs displayed four copies of *TERT*; while 2/22 (9%) FTCs, 4/29 (13.8%) HCCs, 0/22 (0%) MTCs, and 2/4 (50%) PDTCs/ATCs harbored three copies of *TERT*. In addition, one HCC and one PDTC/ATC with 3 copies of *TERT* also harbored the *TERT* −124 C>T mutation (65). A 2018 study, limited to follicular tumors, found three or more copies of *TERT* in 6/77 (7.8%) of FTCs, 2/43 (4.7%) of follicular adenomas, and 4/19 (21%) of follicular tumors of uncertain malignant potential (42). In a 2016 study of MTC, 5/42 (11.9%) had three copies of *TERT*, and no normal thyroid tissue showed copy number variations. In all five tumors with copy number amplifications, *TERT* expression and telomerase activity was also positive (16). Lastly, the comprehensive TCGA study of ~500 PTCs from multiple institutions showed 5p arm copy number gains in 21/507 (4%). In this study, 5p gain correlated with advanced age, worse pathological stage, extrathyroidal extension, and increased tumor size (22).

## Discussion

In summary, *TERT* is regulated by a variety of mechanisms, indicative of the complex regulatory effort cells exert to control telomerase activity. The majority of malignancies reactivate *TERT* expression, which in turn activates telomerase, to allow for continued proliferation. *TERT* reactivation is important for cancer cells, as cancer cells have significantly shorter telomeres. Shortened telomeres are found in PTC, FTC, HCC, and MTC compared to normal thyroid tissue and benign thyroid nodules (60, 62, 66–68). In PTC tumors, short telomeres were significantly correlated with *TERT* promoter mutations and older age (69).

In this review, we have summarized the current knowledge of *TERT* regulation, including *TERT* promoter mutations, CpG promoter methylation, histone tail modifications, copy number amplifications, and alternative splicing. Furthermore, we have described the allele-specific effects of the *TERT* mutation on transcriptional activator binding, promoter methylation, histone marks, and *TERT* expression. While all of these regulatory strategies are altered in thyroid cancer, and may play a significant role in *TERT* activation, these altered regulatory mechanisms have not yet been leveraged clinically.

*TERT* mRNA expression has been proposed as a prognostic marker, independent of the *TERT* promoter mutation. A study in PTC found a subset of wildtype *TERT* tumors with high *TERT* expression, which correlated with a higher recurrence rate (70). However, other studies have shown that TERT immunohistochemistry (IHC), or measurement of protein expression is not a useful clinical tool for prognostication. A study in FTC showed no correlation between *TERT* mRNA expression and TERT immunoreactivity (71), and a study in PTC showed no correlation with TERT IHC and clinicopathological traits (72).

Presence of the *TERT* promoter mutation, however, may be promising for future use to guide clinical decision making and treatment. The presence of the *TERT* promoter mutation is indicative of worse clinicopathological factors in thyroid cancer. The thyroid research field has recently begun to embrace the use of molecular markers to help in making clinical decisions. For example, post-operative screening for the *TERT* promoter mutation in follicular tumors of uncertain malignant potential (FT-UMP) was found to inform follow-up and treatment, as the *TERT* mutation was a predictive marker of distant metastases (73). Further, current molecular testing does include the *TERT* promoter mutations in some platforms, including ThyroSeq and ThyGenX. Current guidelines, however, do not support using the *TERT* mutation status for initial risk stratification (74). In the new American Association of Endocrine Surgeons Guidelines for the Definitive Surgical Management of Thyroid Disease in Adults, there is acknowledgment of inclusion of the *TERT* promoter mutation in assessment of the overall mutational burden in thyroid cancers (74). But for many molecular markers, the utility has not been tested given the newly published guidelines, nor has there been an incorporation of the new reclassification of the Non-Invasive Follicular Thyroid neoplasm with Papillary-like nuclear features (NIFTP). The 2015 American Thyroid Association Management Guidelines for Adult Patients with Thyroid Nodules and Differentiated Thyroid Cancer listed *TERT*, alone or in combination with *BRAF*, as potentially helpful to risk stratify patients in conjunction with other clinicopathological risk factors. However, this was considered a weak recommendation with low-quality evidence only (75).

In conclusion, we know much about telomerase regulation. Capitalizing upon our deep understanding of these regulatory mechanisms may lead to crucial and needed therapeutic options in the future. Nevertheless, major gaps in our understanding of telomerase regulation remain.

Understanding how *TERT* splicing is regulated is essential to a better understanding of the activation of telomerase. Characterization of *TERT* promoter methylation as well as ETS transcriptional activator binding in specific thyroid cancer subtypes, especially in PDTC and ATC, may lead to potential treatment options for these fatal diseases. Additionally, the study of chromosomal architecture surrounding *TERT*, and the effect of the *TERT* mutation on 3D chromatin structure in thyroid cancer have yet to be explored. One aspect that remains to be fully understood is how the higher levels of *TERT* expression and activity found in *TERT*-mutant cells provides a proliferative or other competitive advantage, since cancer cells typically have activated *TERT* expression to overcome the Hayflick limit long before *TERT* promoter mutations arise. Further understanding of how cancer cells that do not harbor the *TERT* mutation activate *TERT* through various regulatory methods still needs to be elucidated. Further studies are needed to determine the true utility of the *TERT* mutation status in a clinical setting and, how best to target *TERT* regulation.

## Author Contributions

BM, CU, and MZ designed, wrote, and edited the manuscript. All authors contributed to the article and approved the submitted version.

## Conflict of Interest

The authors declare that the research was conducted in the absence of any commercial or financial relationships that could be construed as a potential conflict of interest. The reviewer KB declared a shared affiliation, with no collaboration, with the authors, BM and CU to the handling editor at the time of the review.

## Abbreviations

ALT: alternative lengthening of telomeres
ATC: anaplastic thyroid cancer
ATRX: ATRX chromatin remodeler
CpG: cytosine-guanine dinucleotides
DAXX: death domain associated protein
DTC: differentiated thyroid cancer
ETV5: ETS variant 5
FISH: fluorescence *in situ* hybridization
FTC: follicular thyroid cancer
GABPA: GA binding protein transcription factor subunit alpha
HCC: Hürthle cell carcinoma
MAPK: mitogen-activated protein kinase
MTC: medullary thyroid cancer
PDTC: poorly differentiated thyroid cancer
PRC: polycomb repressor complex
PTC: papillary thyroid cancer
TERT: telomerase reverse transcriptase
TR: telomerase RNA
TSS: transcription start site.
